# Supplementary material for: A universal fluorescence-based toolkit for real-time quantification of DNA and RNA nuclease activity
Source: Sci Rep. 2019 Jun 20;9:8853. doi: 10.1038/s41598-019-45356-z (PMC6586798; doi:10.1038/s41598-019-45356-z)
Supplement: Supplementary file 1 — Supplementary material [file 41598_2019_45356_MOESM1_ESM.pdf]

A universal fluorescence-based toolkit for real-time quantification of DNA and RNA nuclease activity

Emily C. Sheppard[1*], Sally Rogers[1*], Nicholas J. Harmer[1] and Richard Chahwan[1,2 #]

[1] *Living Systems Institute, University of Exeter, Stocker Road, Exeter EX4 4QD, UK*
[2] *Institute of Experimental Immunology, University of Zurich, 8057 Zurich, CH*

Supplementary materials

In addition to converting the raw fluorescence values to base pairs of DNA substrate, it is possible to study nuclease activity in terms of digestion of substrate molarity based on the known molarities in the substrate titrations, both plasmid and oligonucleotide (Fig. S1a and c). The linear increase in the in the rate of DNase I-mediated digestion indicates that this is a first order reaction dependent on the quantity of available DNA (Fig. S1b and d).

To test the capabilities of the BITEG and streptavidin combination on protecting the DNA substrates from resection, we compared the activities of both a 3' and 5' exonuclease on different combinations of BITEG and streptavidin. As intended, the combinatorial effect inhibits extensive resection, and approximately 15-20% resection is observed, likely due to impurities in the oligonucleotides. The absence of either BITEG or streptavidin, however, results in resection at a very similar rate as the unmodified substrate (Fig. S2b-c). Unexpectedly, the total resection on a BITEG-blocked terminal end to reveal a ssDNA product is only observed in the absence of streptavidin, while it appears that ExoIII is unable to resect completely, potentially as a result of steric hindrance imposed by the streptavidin molecule.

Supplementary Figure Legends

Figure S1: Fluorescence units can be converted to nM concentrations of DNA substrate

a, Plasmid titration in the presence of 50 nM DNase I. **b,** Calculated the maximum rate of resection of increasing concentrations of plasmid based on the maximum gradients in (a). **c,** Oligonucleotide titration in the presence of 50 nM DNase I. **d,** Calculated the maximum rate of increasing concentrations of an 80-mer dsDNA substrate based on the maximum gradients in (c).

Figure S2: BITEG alone is insufficient for preventing nuclease resection.

a, Stick and ball figures illustrate the substrates and the distinguishing features of the BITEG and streptavidin modifications. **b,** Treated a range of substrates with and without the BITEG and streptavidin modifications in the presence of 12 nM T7 Exo. **c,** Treated a range of substrates with and without BITEG and streptavidin modifications in the presence of 10 nM ExoIII. **d,** Presence of streptavidin prevents total resection of the substrate. **e,** Oligonucleotide titration treated with 10 nM ExoIII. **f,** Calculated the rate of resection according to the maximum gradients in (e) for each dilution of substrate. Error bars represent SEM; n=3 in all cases.

Figure S3: Control experiment for RNAase A nuclease activity

a, RNAase A activity against 40-mer double stranded RNA (RR40 + RNAase A; yellow) compared to no enzyme control (RR40; red) and RNAase A activity against 20-mer double stranded RNA (RR20 + RNAase A; light blue) compared to no enzyme control (RR20; blue). **b,** RNAase A activity against 40-mer DNA:RNA hybrid (DR40 + RNAase A; yellow) compared to no enzyme control (DR40; red) and RNAase A activity against 20-mer DNA:RNA hybrid (DR20 + RNAase A; light blue) compared to no enzyme control (RR20; blue). **c,** RNAase A activity against 40-mer double stranded DNA (DD40 + RNAase A; yellow) compared to no enzyme control (DD40; red).

Figure S4: Control experiment for RNAase H nuclease activity.

a, RNAase H activity against 40-mer single stranded RNA (R40 + RNAase H; yellow) compared to no enzyme control (R40; red) and RNAase H activity against 20-mer single stranded RNA (R20 + RNAase H; light blue) compared to no enzyme control (R20; blue). **b,** RNAase H activity against 40-mer double stranded RNA (RR40 + RNAase H; yellow) compared to no enzyme control (RR40; red) and RNAase H activity against 20-mer double stranded RNA (RR20 + RNAase H; light blue) compared to no enzyme control (RR20; blue). **c,** RNAase A activity against 40-mer double stranded DNA (DD40 + RNAase H; yellow) compared to no enzyme control (DD40; red).

Table S1: Oligonucleotides and their respective stick and ball illustrations

Oligomers	Illustration	Codes
S		RCOL556 / RCOL557
${\cdot}S^{*}$		RCOL621/ RCOL626
S^{*}		RCOL621/ RCOL557
${}_{+2}S^{*}$		RCOL621 / RCOL622
${}_{+4}S^{*}$		RCOL621 / RCOL624
${}^{*}S{\cdot}$		RCOL616 / RCOL620
${}^{*}S$		RCOL616 / RCOL557
${}^{*}S_{+2}$		RCOL616 / RCOL617
${}^{*}S_{+10}$		RCOL616 / RCOL618
${}^{*}S_{+20}$		RCOL616 / RCOL619
${}_{G>A}S^{*}$		RCOL621 / RCOL715
${\cdot}S$		RCOL556 / RCOL626
${\cdot}S^{C>T}$		RCOL714 / RCOL626
${}^{*}S_{G>A}$		RCOL616 / RCOL715
$S{\cdot}$		RCOL556 / RCOL620
${}^{C>T}S{\cdot}$		RCOL714 / RCOL620
${\cdot}S^{4M}$		RCOL710 / RCOL626
${\cdot}S^{1M}$		RCOL711 / RCOL626
${}^{4M}S{\cdot}$		RCOL710 / RCOL620
${}^{1M}S{\cdot}$		RCOL711 / RCOL620
${\cdot}S^{N^{*}}$		RCOL648 / RCOL649 / RCOL626
${\cdot}S^{G^{*}}$		RCOL648 / RCOL650 / RCOL626
${}^{*N}S{\cdot}$		RCOL645 / RCOL646 / RCOL620
${}^{*G}S{\cdot}$		RCOL645 / RCOL647 / RCOL620
60 bp ladder (S_{60})		RCOL556 / RCOL611
40 bp ladder (S_{40})		RCOL556 / RCOL610
20 bp ladder (S_{20})		RCOL556 / RCOL609
KF 20mer overhang (${}^{60}S$)		RCOL558 / RCOL557
KF 40mer overhang (${}^{40}S$)		RCOL560 / RCOL557
KF 60mer overhang (${}^{20}S$)		RCOL562 / RCOL557
R_{40}		RCOL739
R_{20}		RCOL738
RD_{40} (RNA/DNA)		RCOL739 / RCOL610
RD_{20} (RNA/DNA)		RCOL738 / RCOL609

Table S2: Sequence of the toolkit substrates.

Oligo code	Sequence (5' –3')
RCOL556	GATGGTTTGTTGGTCTATTACTACTTGGAGCTTGTATGATTCGAAACCTTGGAGTACTTGCCTACTTGGAGTGAACTTAG
RCOL557	CTAAGTTCACTCCAAGTAGGCAAGTACTCCAAGGTTTCGAATCATACAAGCTCCAAGTAGTAATAGACCAACAAACCATC
RCOL621	GATGGTTTGTTGGTCTATTACTACTTGGAGCTTGTATGATTCGAAACCTTGGAGTACTTGCCTACTTGGAGTGAACTTAG -BITEG*
RCOL626	CTAAGTTCACTCCAAGTAGGCAAGTACTCCAAGGTTTCGAATCATACAAGCTCCAAGTAGTAATAGACCAACAAACCATC-BITEG
RCOL622	CTAAGTTCACTCCAAGTAGGCAAGTACTCCAAGGTTTCGAATCATACAAGCTCCAAGTAGTAATAGACCAACAAACCATC TT
RCOL624	CTAAGTTCACTCCAAGTAGGCAAGTACTCCAAGGTTTCGAATCATACAAGCTCCAAGTAGTAATAGACCAACAAACCATC TTTT
RCOL616	BITEG-GATGGTTTGTTGGTCTATTACTACTTGGAGCTTGTATGATTCGAAACCTTGGAGTACTTGCCTACTTGGAGTGAACTTAG
RCOL620	BITEG-CTAAGTTCACTCCAAGTAGGCAAGTACTCCAAGGTTTCGAATCATACAAGCTCCAAGTAGTAATAGACCAACAAACCATC
RCOL617	TTCTAAGTTCACTCCAAGTAGGCAAGTACTCCAAGGTTTCGAATCATACAAGCTCCAAGTAGTAATAGACCAACAAACCATC
RCOL618	TTTTTTTTTTCTAAGTTCACTCCAAGTAGGCAAGTACTCCAAGGTTTCGAATCATACAAGCTCCAAGTAGTAATAGACCAACAAACCATC
RCOL619	TTTTTTTTTTTTTTTTTTTT CTAAGTTCACTCCAAGTAGGCAAGTACTCCAAGGTTTCGAATCATACAAGCTCCAAGTAGTAATAGACCAACAAACCATC
RCOL714	GATGGTTTGTTGGT **T**TATTACTACTTGGAG **T**TTGTATGATT **T**GAAACCTTGGAGTACTTG **T**CTACTTGGAGTGAACTTAG **
RCOL715	CTAAGTTCACTCCAAGTAG **A**CAAGTACTCCAAGGTTTC **A**AATCATACAA **A**CTCCAAGTAGTAATA **A**ACCAACAAACCATC ***
RCOL710	GATGGTTTGTTGGT **C**TATTACTACTTGGAG **C**TTGTATGATT **C**GAAACCTTGGAGTACTTG **C**CTACTTGGAGTGAACTTAG ****
RCOL711	GATGGTTTGTTGGT CTATTACTACTTGGAG CTTGTATGATT **C**GAAACCTTGGAGTACTTG CCTACTTGGAGTGAACTTAG ****
RCOL648	GTGAACTTAG-BITEG
RCOL649	GATGGTTTGTTGGTCTATTACTACTTGGAGCTTGTATGATTCGAAACCTTGGAGTACTTGCCTACTTGGA
RCOL650	GATGGTTTGTTGGTCTATTACTACTTGGAGCTTGTATGATTCGAAACCTTGGAGTACTTGCCTACTTGG
RCOL645	BITEG-GATGGTTTGT
RCOL646	TGGTCTATTACTACTTGGAGCTTGTATGATTCGAAACCTTGGAGTACTTGCCTACTTGGAGTGAACTTAG
RCOL647	GGTCTATTACTACTTGGAGCTTGTATGATTCGAAACCTTGGAGTACTTGCCTACTTGGAGTGAACTTAG
RCOL611	CTAAGTTCACTCCAAGTAGGCAAGTACTCCAAGGTTTCGAATCATACAAGCTCCAAGTAG
RCOL610	CTAAGTTCACTCCAAGTAGGCAAGTACTCCAAGGTTTCGA
RCOL609	CTAAGTTCACTCCAAGTAGG
RCOL562	GATGGTTTGTTGGTCTATTA
RCOL560	GATGGTTTGTTGGTCTATTACTACTTGGAGCTTGTATGAT
RCOL558	GATGGTTTGTTGGTCTATTACTACTTGGAGCTTGTATGATTCGAAACCTTGGAGTACTTG
RCOL739	TCGAAACCTTGGAGTACTTGCCTACTTGGAGTGAACTTAG
RCOL738	CCTACTTGGAGTGAACTTAG

- * BITEG represents the biotin-TEG modification
- **T bases in bold indicate sites of C>T base changes to generate a mismatched substrate
- ***A bases in bold indicate sites of G>A base changes to generate a mismatched substrate
- ****C bases in bold indicate sites of methylcytosine bases.

Figure S1

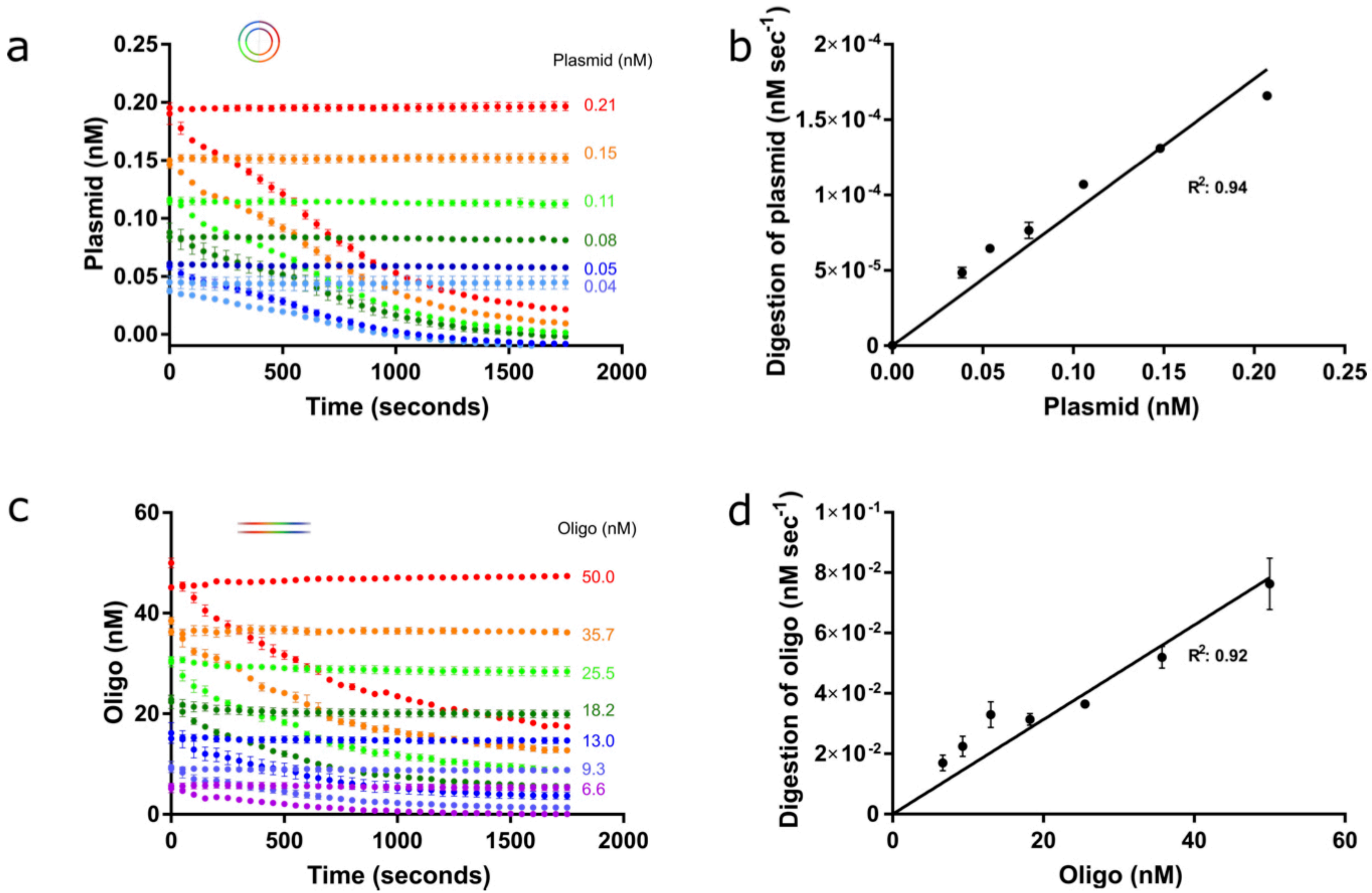
a
Plasmid (nM)
0.21
0.15
0.11
0.08
0.05
0.04
Plasmid (nM)
Time (seconds)
b
Digestion of plasmid (nM sec^{-1})
Plasmid (nM)
R^2: 0.94
c
Oligo (nM)
50.0
35.7
25.5
18.2
13.0
9.3
6.6
Oligo (nM)
Time (seconds)
d
Digestion of oligo (nM sec^{-1})
Oligo (nM)
R^2: 0.92

Figure S2

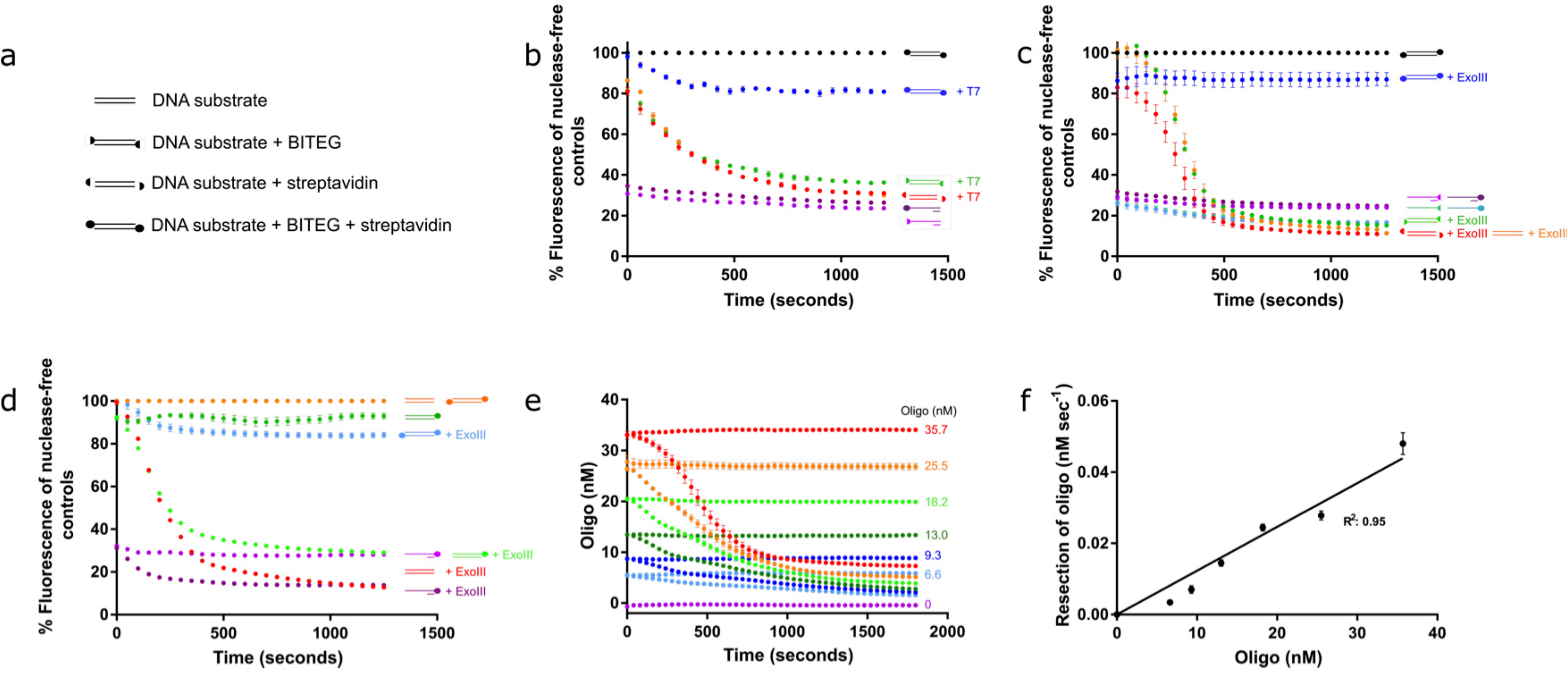

a
DNA substrate
DNA substrate + BITEG
DNA substrate + streptavidin
DNA substrate + BITEG + streptavidin
b
% Fluorescence of nuclease-free controls
Time (seconds)
+ T7
+ T7
+ T7
c
% Fluorescence of nuclease-free controls
Time (seconds)
+ ExoIII
+ ExoIII
+ ExoIII
+ ExoIII
d
% Fluorescence of nuclease-free controls
Time (seconds)
+ ExoIII
+ ExoIII
+ ExoIII
+ ExoIII
e
Oligo (nM)
Time (seconds)
35.7
25.5
18.2
13.0
9.3
6.6
0
f
Resection of oligo (nM sec-1)
Oligo (nM)
R2: 0.95

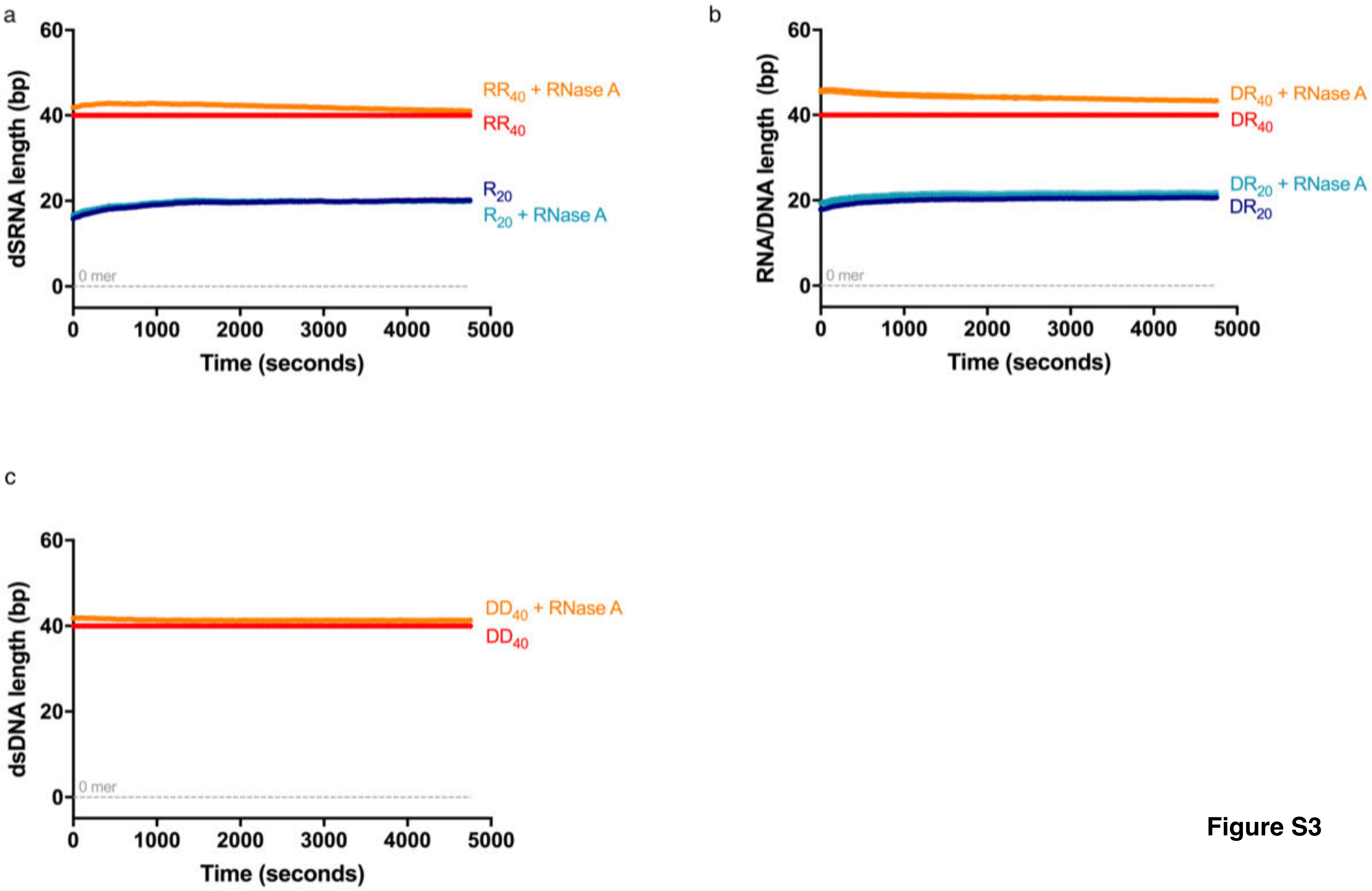
a
dSRNA length (bp)
RR40 + RNase A
RR40
R20
R20 + RNase A
0 mer
Time (seconds)
b
RNA/DNA length (bp)
DR40 + RNase A
DR40
DR20 + RNase A
DR20
0 mer
Time (seconds)
c
dsDNA length (bp)
DD40 + RNase A
DD40
0 mer
Time (seconds)

Figure S3

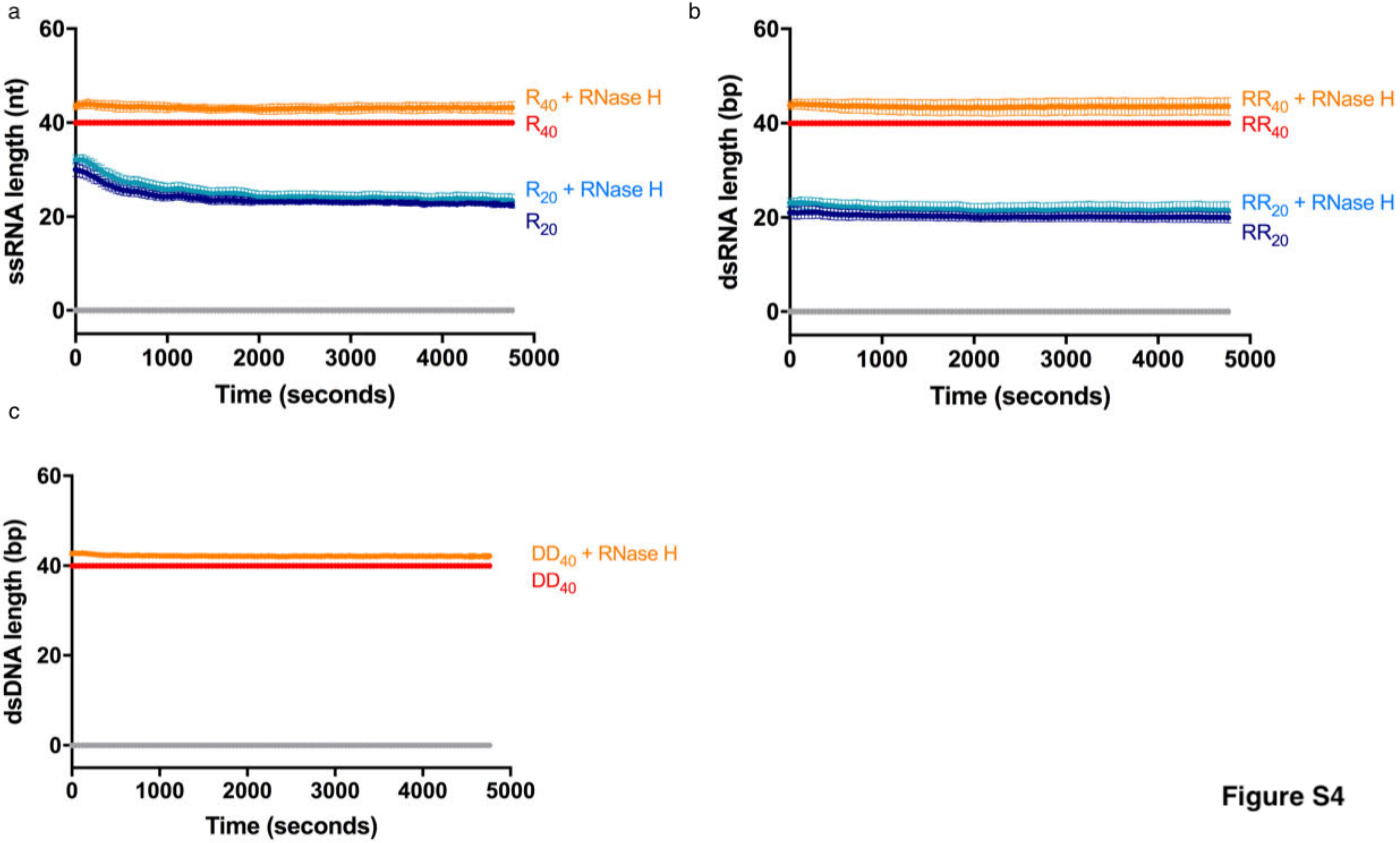
a
ssRNA length (nt)
60
40
20
0
0
1000
2000
3000
4000
5000
Time (seconds)
R40 + RNase H
R40
R20 + RNase H
R20
b
dsRNA length (bp)
60
40
20
0
0
1000
2000
3000
4000
5000
Time (seconds)
RR40 + RNase H
RR40
RR20 + RNase H
RR20
c
dsDNA length (bp)
60
40
20
0
0
1000
2000
3000
4000
5000
Time (seconds)
DD40 + RNase H
DD40

Figure S4

Supplementary methods

<u>Analysis of nuclease rates</u>

Part 1: Converting fluorescence units to length of duplex DNA

Raw data from a DNase I-mediated digestion of 80-bp dsDNA:

	A	B	C	D	E	F	G	H	I	J	K	L	M
1	time (seconds	50 nM 556/557 + 150 nM DNase			50 nM 556/557 + 83.3 nM DNase			50 nM 556/557 + 46.3 nM DNase			50 nM 556/557 +25.7 nM DNase		
2	0	42787	43683	42130	40609	42842	44042	42295	42736	47628	46719	49808	51063
3	50	40757	39385	38979	37308	39522	41283	39448	41389	46005	44355	46858	48846
4	100	37483	36812	36073	35031	36781	38331	37682	38670	44554	41465	45987	47093
5	150	32137	33157	33352	32724	34693	36174	36433	37434	41459	40017	44375	45756
6	200	28075	29946	30649	30462	32873	33737	34455	36048	40235	38105	42396	44105
7	250	24964	26768	27754	27688	30529	31083	32742	34324	38592	37477	40612	42363
8	300	22726	23968	24953	25312	28023	28268	31855	32324	37080	35953	38435	40533
9	350	20252	21876	22872	23555	26322	26369	29022	31343	35405	34743	37466	39434
10	400	18086	20001	20830	21549	24334	24580	27391	29846	33412	33012	35307	38685
11	450	16688	18352	19255	19929	22615	22845	26000	27305	32984	31798	34879	35747
12	500	15431	16947	18007	18882	21514	21568	24908	26887	31352	31152	34678	35566
13	550	14253	15804	16870	17726	20263	20428	24722	25878	29869	29787	31929	33868
14	600	13179	14692	15844	16781	19186	19319	23461	24555	28024	29013	31836	32310
15	650	12166	13756	14816	15934	18144	18281	21344	23640	26847	27759	30115	31076
16	700	11349	12964	13892	15077	17181	17307	21750	23208	26156	27037	29049	30635
17	750	10517	12144	13094	14453	16459	16659	21227	22584	24649	26005	28035	29912
18	800	9887	11375	12272	13636	15692	15833	20428	21835	23987	25513	26854	28960
19	850	9203	10751	11647	13084	15036	15180	19641	21129	22656	25570	26260	27699
20	900	8639	10153	10955	12518	14441	14634	19089	20788	22059	25057	26210	26709
21	950	8117	9554	10267	11860	13791	14049	18237	19763	21635	24387	26184	26547
22	1000	7665	8998	9697	11341	13214	13448	17793	19064	21272	23815	25281	26049
23	1050	7164	8480	9131	10757	12611	12783	17644	18770	20501	23211	25253	25486
24	1100	6820	8050	8599	10252	12021	12139	17522	18392	19783	22717	25505	24958
25	1150	6420	7620	8173	9875	11610	11732	17084	18051	19390	22313	24947	24565
26	1200	6115	7230	7786	9523	11204	11379	16669	17565	19212	22155	24441	24214
27	1250	5766	6927	7411	9151	10797	10877	16326	17239	18773	21833	23936	24055
28	1300	5459	6540	7048	8754	10316	10465	15893	16559	18164	21304	22926	24551
29	1350	5171	6229	6692	8429	9919	10051	15518	16490	17640	21368	22429	24484
30	1400	4960	5980	6379	8105	9605	9740	15276	16452	17480	21027	22246	23917
31	1450	4727	5743	6161	7812	9271	9367	14944	15963	17177	20604	21850	23405
32	1500	4576	5490	5873	7506	8906	9067	14548	15871	16896	20299	21613	22894
33	1550	4364	5255	5661	7270	8656	8781	14355	15565	16625	20083	21297	22624
34	1600	4165	5062	5411	6974	8355	8500	14132	15261	16390	19766	20642	22323
35	1650	3985	4856	5179	6679	8112	8190	14020	14763	15961	19599	20319	21999
36	1700	3827	4628	4926	6440	7783	7896	13906	14578	15508	19517	20229	21907
37	1750	3649	4429	4730	6318	7573	7649	13735	14160	15389	19197	20239	21770
38	1800	3519	4282	4534	6019	7269	7380	13531	14002	15208	19063	20084	21489
39	1850	3418	4159	4421	5918	7108	7210	13339	13720	14935	19073	19895	21530

	N	O	P	Q	R	S	T	U	V	W	X	Y
1	50 nM 556/557 +14.3 nM DNase			50 nM 556/557 + 7.9 nM DNase			50 nM 556/557 + 4.4 nM DNase			50 nM 556/557 + 0 nM DNase		
2	45963	47348	50846	47411	48675	50602	48824	49710	54188	50158	50160	53034
3	43622	45782	47517	45986	46976	49190	47347	48021	52248	48555	47841	51812
4	41131	44705	46117	44493	45900	47840	45684	47010	50228	47669	47129	50390
5	39971	43011	44205	43130	44742	46410	44598	46351	48887	46769	46945	49775
6	38944	40888	42762	42223	44260	45478	43788	45648	47771	45944	47147	48908
7	38299	40429	41767	41403	43270	44742	42854	45016	46873	45116	46551	48158
8	36447	38700	40003	40835	42489	44107	42240	44412	46425	44619	45680	47555
9	35719	38371	39533	39438	41533	43485	41616	43495	45584	44313	44618	46873
10	34402	36948	38198	38968	40622	42671	40938	42714	44719	43685	43838	46071
11	33925	35953	36956	38095	40084	41828	40204	41816	44193	43297	43405	45605
12	34308	36015	36219	37477	39251	40943	39651	41218	43496	42821	42752	45164
13	32884	35408	35309	36960	38611	40608	39301	40888	42891	42468	42247	44503
14	31935	34218	35326	36517	37718	40008	38963	40507	42556	41921	41995	44273
15	30401	32634	34155	36519	37733	39470	38675	39898	41934	41696	41778	43964
16	30377	32791	33521	35888	37565	38448	38354	39291	41290	41246	41464	43626
17	30063	32072	33034	35514	37391	38363	37905	38749	40821	40803	41150	43140
18	29256	31149	32294	34873	36807	37872	37644	38430	40397	40766	41092	43081
19	28763	30790	31857	34327	36063	37344	37079	38172	39911	40491	40783	42828
20	28178	30324	31346	33776	35389	36755	36583	38056	39269	40116	40516	42435
21	27723	30179	30736	33464	34483	36421	36245	37630	39031	39951	40363	42149
22	27429	30042	30274	33104	33888	36356	35830	37072	38758	39632	40019	41888
23	27344	30138	29757	32686	33691	36329	35588	36611	38425	39417	39801	41637
24	27311	29770	30028	32337	33431	36058	35387	36353	38046	39305	39502	41382
25	27442	29330	29925	31963	33164	35664	35161	36259	37619	39066	39435	41231
26	26979	28938	29424	31809	32944	35400	34801	35990	37394	38920	39321	41028
27	26605	28621	29070	31560	32544	34960	34463	35876	37037	38748	39039	40826
28	26016	27884	28683	31201	32257	34653	34204	35562	36742	38505	38940	40675
29	25527	27346	28179	30895	31953	33964	33897	35247	36596	38238	38728	40361
30	25264	26965	27859	30758	31716	33727	33721	34986	36311	38148	38476	40175
31	25224	26795	27692	30566	31587	33456	33401	34898	36168	38057	38333	40184
32	24799	26376	27378	30348	31323	33213	33096	34595	35964	37769	38301	39878
33	24410	26107	27136	30251	31252	32963	32888	34538	35738	37702	38031	39725
34	24276	26140	26969	29989	31005	32825	32841	34251	35681	37466	37919	39513
35	24203	25927	26449	29836	30981	32712	32854	34016	35507	37290	37592	39410
36	24197	25724	26269	29775	31038	32611	32651	33838	35316	37164	37563	39269
37	23929	25468	25947	29703	30951	32443	32230	33539	35051	37113	37482	39185
38	23704	25163	25720	29020	30682	32259	31972	33349	34770	36902	37429	39004
39	23450	24950	25704	29279	30659	32112	32026	33256	34575	36718	37242	38862

	Z	AA	AB	AC	AD	AE	AF	AG	AH	AI	AJ	AK	AL	AM	AN
1	50 nM 556/609			50 nM 556/610			50 nM 556/611			50 nM 556			0 nM DNA		
2	17663	16668	17470	20366	19469	19255	36133	33366	28662	10764	10293	9135	168	242	197
3	16817	16008	16713	19614	18233	18403	35510	31752	28191	10164	9613	8582	166	267	197
4	16073	15267	15917	19024	17805	17449	33883	29954	26645	9570	8953	8174	162	351	220
5	15655	14699	15217	18343	17355	16806	32869	29419	25839	9127	8392	7940	162	382	190
6	15075	14182	14634	17646	16930	16070	31769	28329	25126	8737	7857	7550	155	382	190
7	14469	13714	13996	17012	16377	15609	30707	27377	24436	8319	7426	7223	149	395	179
8	14172	13456	13881	16506	15898	14976	29520	26562	23915	7939	6887	6866	159	378	195
9	13621	12986	13407	15886	15417	14578	28307	25553	23499	7590	6621	6571	150	356	180
10	13270	12520	12885	15559	14993	14162	27207	24577	22850	7285	6427	6268	144	344	171
11	13055	12341	12701	15068	14510	13912	26554	23786	22521	7004	6160	6015	149	350	176
12	12606	11970	12114	14596	14262	13544	25933	23268	21981	6753	6003	5833	144	332	182
13	12233	11665	11702	14194	13942	13273	25197	22501	21720	6560	5659	5658	137	314	159
14	12060	11460	11330	13795	13748	13158	24744	21837	21647	6456	5449	5488	143	352	169
15	11946	11236	11111	13300	13638	13004	24362	21530	21561	6330	5290	5319	136	313	156
16	11600	11091	10888	12922	13423	12805	24083	21368	21517	6170	5083	5199	139	297	168
17	11423	10961	10759	12575	13254	12554	23836	21244	21389	6098	4843	5038	134	291	156
18	11226	10843	10624	12349	13036	12413	23662	21123	21175	5958	4722	4929	136	284	154
19	11084	10810	10517	12358	12824	12188	23544	20756	20960	5843	4589	4731	140	268	150
20	10854	10667	10399	12147	12601	11961	23301	20537	20781	5771	4451	4609	133	266	155
21	10460	10497	10251	11970	12418	11782	23133	20413	20618	5591	4334	4524	133	254	677
22	10433	10376	10144	11774	12228	11617	22802	20126	20461	5487	4203	4429	133	242	154
23	10341	10253	10034	11600	11981	11439	22638	19916	20274	5410	4120	4338	132	168	146
24	10215	10185	9890	11344	11836	11308	22491	19539	20108	5321	4037	4243	137	212	156
25	10127	10048	9772	11184	11620	11191	22256	19472	19986	5270	3961	4209	137	227	146
26	10035	10013	9725	11016	11446	11023	22102	19216	19709	5163	3877	4104	132	251	148
27	9919	9922	9664	10922	11394	10888	22026	18911	19655	5125	3793	4016	133	247	143
28	9846	9811	9532	10798	11189	10779	21912	18886	19505	5029	3705	4007	127	186	146
29	9794	9706	9477	10698	11044	10643	21637	18647	19396	4975	3644	3953	130	157	143
30	9685	9601	9371	10568	10927	10570	21481	18440	19281	4936	3604	3855	125	189	150
31	9656	9569	9329	10428	10876	10442	21373	18336	19122	4883	3539	3785	128	198	148
32	9547	9484	9235	10322	10743	10296	21220	18186	18983	4800	3503	3732	127	151	149
33	9507	9386	9154	10269	10541	10175	21079	17945	18934	4758	3440	3715	131	141	149
34	9424	9270	9085	10134	10485	10115	20989	17889	18843	4728	3394	3671	123	130	144
35	9292	9201	9016	10081	10395	9998	20772	17683	18750	4695	3348	3644	123	132	151
36	9250	9147	8922	10010	10277	9925	20723	17605	18675	4663	3314	3580	132	138	150
37	9191	9067	8821	9889	10207	9730	20569	17344	18599	4613	3296	3557	121	134	148
38	9139	8983	8765	9789	10078	9683	20446	17338	18415	4571	3267	3515	132	138	150
39	9078	8965	8811	9760	9947	9594	20394	17253	18414	4526	3197	3515	121	134	148

First the raw data was converted from the standard curve to a measurement of length of duplex DNA. To do this, the average fluorescence for each data set was calculated, and each column was labelled with the length of dsDNA they each represent. The standard curve was produced from the following oligonucleotides:

80 bp = S (556/557)

60 bp = S_{60} (556/611)

40 bp = S_{40} (556/610)

20 bp = S_{20} (556/609)

The latter assays which incorporated substrate variants were normalised as a percentage to their controls (no enzyme) before the standard curve was generated.

	A	B	C	D	E	F	G	H	I	J	K	L	M
42	time (seconds)	50 nM 556/557 + 0 nM DNase			50 nM 556/609 + 0 nM DNase			50 nM 556/610 + 0 nM Dnase			50 nM 556/611 + 0 nM Dnase		
43	0	50158	50160	53034	17663	16668	17470	20366	19469	19255	36133	33366	28662
44	50	48555	47841	51812	16817	16008	16713	19614	18233	18403	35510	31752	28191
45	100	47669	47129	50390	16073	15267	15917	19024	17805	17449	33883	29954	26645
46	150	46769	46945	49775	15655	14699	15217	18343	17355	16806	32869	29419	25839
47	200	45944	47147	48908	15075	14182	14634	17646	16930	16070	31769	28329	25126
48	250	45116	46551	48158	14469	13714	13996	17012	16377	15609	30707	27377	24436
49	300	44619	45680	47555	14172	13456	13881	16506	15898	14976	29520	26562	23915
50	350	44313	44618	46873	13621	12986	13407	15886	15417	14578	28307	25553	23499
51	400	43685	43838	46071	13270	12520	12885	15559	14993	14162	27207	24577	22850
52	450	43297	43405	45605	13055	12341	12701	15068	14510	13912	26554	23786	22521
53	500	42821	42752	45164	12606	11970	12114	14596	14262	13544	25933	23268	21981
54	550	42468	42247	44503	12233	11665	11702	14194	13942	13273	25197	22501	21720
55	600	41921	41995	44273	12060	11460	11330	13795	13748	13158	24744	21837	21647
56	650	41696	41778	43964	11946	11236	11111	13300	13638	13004	24362	21530	21561
57	700	41246	41464	43626	11600	11091	10888	12922	13423	12805	24083	21368	21517
58	750	40803	41150	43140	11423	10961	10759	12575	13254	12554	23836	21244	21389
59	800	40766	41092	43081	11226	10843	10624	12349	13036	12413	23662	21123	21175
60	850	40491	40783	42828	11084	10810	10517	12358	12824	12188	23544	20756	20960
61	900	40116	40516	42435	10854	10667	10399	12147	12601	11961	23301	20537	20781
62	950	39951	40363	42149	10460	10497	10251	11970	12418	11782	23133	20413	20618
63	1000	39632	40019	41888	10433	10376	10144	11774	12228	11617	22802	20126	20461
64	1050	39417	39801	41637	10341	10253	10034	11600	11981	11439	22638	19916	20274
65	1100	39305	39502	41382	10215	10185	9893	11344	11836	11308	22491	19599	20108
66	1150	39066	39435	41231	10127	10048	9772	11184	11620	11191	22256	19472	19986
67	1200	38920	39321	41028	10035	10013	9725	11016	11446	11023	22102	19216	19709

	N	O	P	Q	R	S	T	U	V	W
42	0 nM DNA				time (seconds)	80	60	40	20	0
43	168	242	197		0	51117.3	32720.3	19696.7	17267	202.333
44	166	267	197		50	49402.7	31817.7	18750	16512.7	210
45	162	351	220		100	48396	30160.7	18092.7	15752.3	244.333
46	162	382	190		150	47829.7	29375.7	17501.3	15190.3	244.667
47	155	382	190		200	47333	28408	16882	14630.3	242.333
48	149	395	179		250	46608.3	27506.7	16332.7	14059.7	241
49	159	378	195		300	45951.3	26665.7	15793.3	13836.3	244
50	150	356	180		350	45268	25786.3	15293.7	13338	228.667
51	144	344	171		400	44531.3	24878	14904.7	12891.7	219.667
52	149	350	176		450	44102.3	24287	14496.7	12699	225
53	144	332	182		500	43579	23727.3	14134	12230	219.333
54	137	314	159		550	43072.7	23139.3	13803	11866.7	203.333
55	143	352	169		600	42729.7	22742.7	13567	11616.7	221.333
56	136	313	156		650	42479.3	22484.3	13314	11431	201.667
57	139	297	168		700	42112	22322.7	13050	11193	201.333
58	134	291	156		750	41697.7	22156.3	12794.3	11047.7	193.667
59	136	284	154		800	41646.3	21986.7	12599.3	10897.7	191.333
60	140	268	150		850	41367.3	21753.3	12456.7	10803.7	186
61	133	266	155		900	41022.3	21539.7	12236.3	10640	184.667
62	133	254	677		950	40821	21388	12056.7	10402.7	354.667
63	133	242	154		1000	40513	21129.7	11873	10317.7	176.333
64	132	168	146		1050	40285	20942.7	11673.3	10209.3	148.667
65	137	212	156		1100	40063	20732.7	11496	10097.7	168.333
66	137	227	146		1150	39910.7	20571.3	11331.7	9982.33	170
67	132	251	148		1200	39756.3	20342.3	11161.7	9924.33	177

Next, we used these averaged values to determine the equation of the line, as indicated in Fig. 1c in the main text. We had to find the values for the slope (m) and intercept (c) in the linear equation y = mx + c.

Equation to calculate the slope (m): "=SLOPE(known_y's, $known_x's)"

E.g., the first equation at 0 seconds is: "=SLOPE(S43:W43,S$42:W$42)"

The "$" locks the known_x's values, as these values remain constant in all iterations of this equation.

Equation to calculate the intercept (c): "=INTERCEPT(known_y's, $known_x's)"

E.g., the first equation at 0 seconds is: "=INTERCEPT(S43:W43,S$42:W$42)"

R	S	T	U	V	W	X	Y	Z
time (seconds)	80	60	40	20	0		slope (m)	intercept (c)
0	51117.3	32720.3	19696.7	17267	202.333		586.417	744.067
50	49402.7	31817.7	18750	16512.7	210		568.452	600.533
100	48396	30160.7	18092.7	15752.3	244.333		553.558	386.867
150	47829.7	29375.7	17501.3	15190.3	244.667		546.777	157.267
200	47333	28408	16882	14630.3	242.333		539.795	-92.6667
250	46608.3	27506.7	16332.7	14059.7	241		530.908	-286.667
300	45951.3	26665.7	15793.3	13836.3	244		521.22	-350.667
350	45268	25786.3	15293.7	13338	228.667		512.635	-522.467
400	44531.3	24878	14904.7	12891.7	219.667		503.048	-636.867
450	44102.3	24287	14496.7	12699	225		496.713	-706.533
500	43579	23727.3	14134	12230	219.333		491.083	-865.4
550	43072.7	23139.3	13803	11866.7	203.333		485.057	-985.267
600	42729.7	22742.7	13567	11616.7	221.333		480.713	-1053.07
650	42479.3	22484.3	13314	11431	201.667		478.043	-1139.67
700	42112	22322.7	13050	11193	201.333		474.755	-1214.4
750	41697.7	22156.3	12794.3	11047.7	193.667		470.583	-1245.4
800	41646.3	21986.7	12599.3	10897.7	191.333		469.995	-1335.53
850	41367.3	21753.3	12456.7	10803.7	186		466.562	-1349.07
900	41022.3	21539.7	12236.3	10640	184.667		462.875	-1390.4
950	40821	21388	12056.7	10402.7	354.667		459.59	-1379
1000	40513	21129.7	11873	10317.7	176.333		457.427	-1495.13
1050	40285	20942.7	11673.3	10209.3	148.667		455.03	-1549.4
1100	40063	20732.7	11496	10097.7	168.333		452.122	-1573.33
1150	39910.7	20571.3	11331.7	9982.33	170		450.352	-1620.87
1200	39756.3	20342.3	11161.7	9924.33	177		447.883	-1643

Using the calculated values for the slope and intercept, it was possible to convert the raw fluorescence values to length of dsDNA. In doing so, we had to solve for ‘x’ in the equation y = mx + c at each time point, where ‘y’ = a raw fluorescence value, ‘m’ = the slope, calculated previously, and ‘c’ = the intercept.

SUM =(E3-$B3)/$A3

	A	B	C	D	E	F	G	H	I	J	K
1					Raw fluorescence values				Converted to length of dsDNA		
2	slope (m)	intercept (c)		time (seconds)	50 nM 556/557 + 150 nM DNase				50 nM 556/557 + 150 nM DNase		
3	586.4167	744.0667		0	42787	43683	42130		=(E3-$B3)	73.22257	70.57428
4	568.4517	600.5333		50	40757	39385	38979		70.64183	68.22826	67.51404
5	553.5583	386.8667		100	37483	36812	36073		67.01396	65.8018	64.4668
6	546.7767	157.2667		150	32137	33157	33352		58.48774	60.35322	60.70986
7	539.795	-92.6667		200	28075	29946	30649		52.18216	55.64829	56.95063
8	530.9083	-286.667		250	24964	26768	27754		47.56126	50.95921	52.8164
9	521.22	-350.667		300	22726	23968	24953		44.27433	46.6572	48.547
10	512.635	-522.467		350	20252	21876	22872		40.52487	43.69282	45.63572
11	503.0483	-636.867		400	18086	20001	20830		37.21882	41.02561	42.67357
12	496.7133	-706.533		450	16688	18352	19255		35.01926	38.36928	40.18723
13	491.0833	-865.4		500	15431	16947	18007		33.18459	36.27164	38.43014
14	485.0567	-985.267		550	14253	15804	16870		31.41544	34.613	36.81068
15	480.7133	-1053.07		600	13179	14692	15844		29.60614	32.75355	35.14999
16	478.0433	-1139.67		650	12166	13756	14816		27.8336	31.15966	33.37703
17	474.755	-1214.4		700	11349	12964	13892		26.46291	29.86467	31.81936
18	470.5833	-1245.4		750	10517	12144	13094		24.99536	28.45277	30.47154
19	469.995	-1335.53		800	9887	11375	12272		23.87798	27.04398	28.95251
20	466.5617	-1349.07		850	9203	10751	11647		22.61666	25.93455	27.85498
21	462.875	-1390.4		900	8639	10153	10955		21.66762	24.93848	26.67113
22	459.59	-1379		950	8117	9554	10267		20.66189	23.78859	25.33998
23	457.4267	-1495.13		1000	7665	8998	9697		20.02536	22.93949	24.4676

This data can then be analysed to calculate maximal reaction rates using the equation ‘x = (y - c) / m) as shown in the above figure.

Part 2: Rate analysis

It is possible to calculate the rate of the reaction by calculating the slope of the curve at its steepest gradient. This is the maximum rate at which the enzyme digests or resects the DNA substrate. Below, the rate at the highest concentration of DNase I (150 nM) and in the absence of DNase I (0 nM DNase I) is shown.

SUM =SLOPE(B5:B9,$A$5:$A$9)

	A	B	C	D	E	F	G
1	time (seconds)	50 nM 556/557 + 150 nM DNase			50 nM 556/557 + 0 nM DNase		
2	0	71.69464	73.22257	70.57428	84.2642	84.26761	89.16857
3	50	70.64183	68.22826	67.51404	84.3598	83.10375	90.08939
4	100	67.01396	65.8018	64.4668	85.41491	84.4394	90.33038
5	150	58.48774	60.35322	60.70986	85.24821	85.5701	90.74589
6	200	52.18216	55.64829	56.95063	85.28546	87.51409	90.77644
7	250	47.56126	50.95921	52.8164	85.51884	88.22176	91.24865
8	300	44.27433	46.6572	48.547	86.27771	88.31332	91.91065
9	350	40.52487	43.69282	45.63572	87.4608	88.05576	92.45461
10	1850	12.57635	14.32644	14.94523	91.22384	92.46141	96.28751
11							
12	Slope	=SLOPE(B5:B9	-0.08462	-0.0771	0.010835	0.011541	0.009103
13							
14							
15	Rate	150 nM Dnase	0 nM Dnase				
16		-0.08766714	0.010835		blank	0.010493	
17		-0.08462378	0.011541				
18		-0.07710382	0.009103				
19							
20	Rate minus average 0 nM Dnase	150 nM Dnase	0 nM DNase				
21		-0.09816023	0.000342				
22		-0.09511687	0.001048				
23		-0.08759691	-0.00139				

The rate for this reaction is calculated for each individual sample. An average of the rate when there is 0 nM DNase I is taken (0.010493) and this is subtracted from each calculation of the slope.

The rate is then divided by the concentration of DNase I in nM. Each value is divided either by 150 (when treated with 150 nM DNase) or 0 (when treated with 0 nM DNase). When divided by 0, the result is 0, as there is no reaction when the enzyme is absent.

The rate is negative, as we are calculating the decrease in florescence over time. However, the rate can be presented in terms of positive integers, and so the negative values are multiplied by -1.

Error is represented by standard error of the mean. The equation for this is: “=STDEV(range)/SQRT(COUNT(range))”.

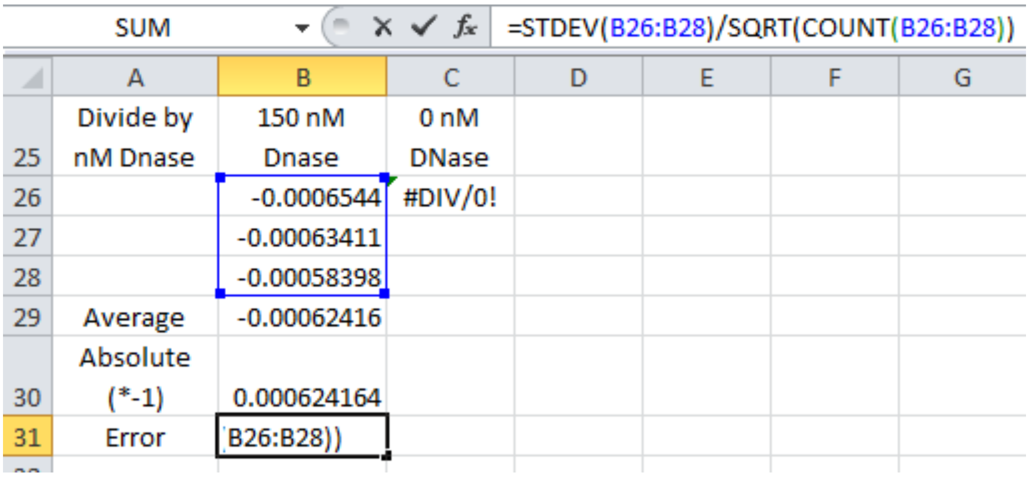

SUM =STDEV(B26:B28)/SQRT(COUNT(B26:B28))

	A	B	C	D	E	F	G
25	Divide by nM Dnase	150 nM Dnase	0 nM DNase				
26		-0.0006544	#DIV/0!				
27		-0.00063411					
28		-0.00058398					
29	Average	-0.00062416					
30	Absolute (*-1)	0.000624164					
31	Error	B26:B28))					
